# Soil transmitted helminth infections in Ghana: a ten year review

**DOI:** 10.11604/pamj.2020.35.131.21069

**Published:** 2020-04-20

**Authors:** Monica Ahiadorme, Emmanuel Morhe

**Affiliations:** 1 Centre for Global Health Research, Juaben Government Hospital, KNUST Kumasi, Ashanti Region, Ghana; 2 University of Health and Allied Sciences, Ho, Ghana

**Keywords:** Prevalence, pattern, ecological zone, soil transmitted helminths, Ghana

## Abstract

For more than a decade, intervention programs have been instituted in Ghana to combat soil transmitted helminth (STHs) infections. Knowledge of the trend of the infection in the country is needed for evaluation and modification of existing control programs to achieve national targets. The objective of this review is to examine the pattern of soil transmitted infections in Ghana between 2009-2018. We searched and reviewed published literature on soil transmitted helminths in Ghana in PubMed, Medline, Google Scholar and Institutional Repositories of Kwame Nkrumah University of Science and Technology, University of Ghana, University of Cape Coast, and University for Development Studies-Tamale. We observed paucity of research work on STHs in Ghana over the period of this review. Twenty-nine studies consisting of 24 published works in peer reviewed journals and five graduate theses were included in the study. Hookworm was the most prevalent of STHs recorded followed by roundworm, threadworm, and whipworm. Pinworm was very rarely reported. These infections were reported from different regions and ecological zones of the country and among children, pregnant women, farmers, food vendors, children in orphanage home and psychiatric institution. Although there is some downward trend over the period, soil transmitted helminths are still prevalent in Ghana. This is an indication of some hope of eventual control and elimination of these diseases in the country if control measures are optimised. Further research particularly population studies into soil transmitted helminths in Ghana is needed.

## Introduction

Soil transmitted helminths (STHs) are parasitic worms that live in the intestine of humans and other animals. The infections are associated with inadequate water supply, poor personal hygiene and environmental sanitation practices. Despite advancement in science and technology, these infections continue to be prevalent in less developed countries of sub-Saharan Africa, Latin America and Asia, where sanitation and hygiene remain major challenges [1, 2]. Worldwide, approximately 2 billion people are infected by STHs with the highest prevalence occurring in sub-Saharan Africa [3]. These infections are associated with high morbidity and mortality. Thus, the World Health Organization targets their elimination. Progressive socio-economic development with adoption of effective control measures offers hope to people living in deprived communities of eventual freedom from STHs [4]. The three most common species of STHs worldwide are roundworm (*Ascaris lumbricoides*), whipworm (*Trichuris trichiura*) and hookworms (*Necator americanus* or *Ancylostoma duodenale*) [5]. Worldwide, roundworm and hookworms infect about 1.2 billion and 740 million people respectively [1, 6]. Threadworm (*Strongyloides stercoralis*), another STH often associated with major morbidity in some settings has caught little research attention [7].

These helminths have complex life cycle, part of which occurs in the soil. The eggs are excreted in faeces of infected individuals and open defecation results in contamination of the soil. Humans become infected through ingestion of eggs or larvae with poorly prepared food or eating with inadequately washed hands. Hookworm infection occurs through active penetration of the skin by matured hookworm larvae when humans come in contact with the infested soil. Soil transmitted helminth infections do not occur directly from person to person or through contact with freshly passed faeces. Usually, the eggs and larvae become mature and infective three weeks after they are passed in faeces into the soil [8]. Populations mostly at risk of acquiring STHs are children, pregnant women and farmers who frequently come in contact with the soil [9]. The worms usually cause asymptomatic chronic infections [10]. Heavy infection is associated with intestinal damage with loss of nutrients resulting in impaired mental and physical growth, anaemia, low resistance to other infections of children and poor birth outcomes of pregnant women [11]. Children and pregnant women are most vulnerable to developing complications because they are in physiological state of high demand for nutrients for growth and development.

World Health Organization targeted STHs for control and prevention to reduce maternal and child morbidity and mortality associated with the infections. The recommended effective and easily available strategies include education on safe and hygienic handling of human faeces, wearing footwear in areas designated for defecation and improving quality of water supply, sanitation, and hygiene (WASH). Other measures have been periodic mass administration of antihelminthic drugs through school-based treatment programs [12, 13]. By adopting these measures, WHO globally target eliminating morbidity due to STHs in children by 2020. In Ghana, there have been sporadic health education and deworming programs in various parts of the country since 1994. This has been done by the Ghana Health Service in collaboration with non-governmental organizations (NGOs) [14, 15]. Following STHs mapping conducted in 2007-2008, nationwide interventions have been instituted to combat the infections [16]. There has been mass prophylactic treatment among school age children. The country has been undergoing rapid urbanization with some improvement in social and economic status of the populace [17, 18]. With these developments, current pattern of STHs in the country is needed for evaluation and modification of existing control programs to achieve national targets. There is paucity of this important information in recent literature. We believe a review of publications would produce results that guide researchers, policy makers and implementers as well as development partners in making targeted and cost-effective decisions on improving control and prevention of STHs in Ghana. This literature review examined prevalence and pattern of soil transmitted infections in Ghana. Specifically, it determined types of soil transmitted helminths, geographical and population distributions, and trend of the infections in Ghana over a ten-year period of 2009-2018.

## Methods

We performed a review of research based published literature on STHs in Ghana in peer-reviewed journals and theses in institutional repositories of universities in the country over a ten year period of January 1^st^, 2009 to December 31^st^, 2018. We employed PRISMA guidelines in the conduct of the study to ensure the review was well carried out [19]. However, we did not register the study as a systematic review. The review covered studies conducted in Ghana; a tropical sub-Saharan African country situated in the central west coast of the continent. Ghana is bordered on the west by La Cote d´Ivoire, north by Burkina Faso, east by Togo and south by the Gulf of Guinea of the Atlantic Ocean. Average annual precipitation recorded in 2014 was 1187 mm with heavy rainfall occurring in the southern and middle zones. The country is geographically divided into six major ecological zones: Sudan Savanna Zone, Wet and Moist Evergreen Forest Zone, Deciduous Forest Zone, Coastal Savanna Zone, Transitional Zone and Guinea Savanna Zone [20].

Ghana has ten administrative regions. The population in 2017 was estimated to be 28.96 million with an estimated gross domestic product (GDP) per capita of 1,632 dollars [21]. Approximately, 23.4% of the population live in poverty [22]. About 85% of children are enrolled in primary, 53% in secondary school and 16% in tertiary. Ghana has 71% adult literacy rate [23]. Whiles about 89% of the country´s population has access to safe water, 23% do not have access to adequate sanitation facilities. Only 15% has improved unshared sanitation facilities [24]. Hence, the country is known to have high prevalence of soil transmitted helminths and other infections associated with poor environmental sanitation such as enteric fever, diarrheal diseases including cholera. The Ministry of Health implemented a policy of antihelminthic treatment of school age children at least once in a year [7]. The implementation of national deworming programs for school children was done by Ghana Health Service in collaboration with Ghana Education Service in partnership with UNICEF and other organizations started in 2007 in 60 districts [16, 25]. Among pregnant women, STHs control policy includes education on worm infestation in pregnancy, the connection between the infection and maternal anaemia, and administration of an antihelminthic drug after the first trimester of pregnancy [26, 27].

**Study materials:** publications arising out of yield research work in Ghana constituted the materials for this study.

**Inclusion/exclusion criteria:** included in this review were studies published in peer reviewed journals and theses from repositories of universities in Ghana. All identified research works on STHs, which were conducted in Ghana between January 1^st^, 2009 and December 31^st^, 2018 that reported the infections in human population were included. Studies conducted before 2009 and after December 2018 were excluded. Only articles published in English language literature were included in the review. Newspaper reports were not included.

**Databases searched:** this was internet-based literature search. Databases searched for published materials were PubMed, Medline and Google Scholar and Institutional Repositories of Kwame Nkrumah University of Science and Technology (KNUST), University of Ghana (UG), University of Cape Coast (UCC) and University for Development Studies (UDS), Tamale.

**Search words or Keywords:** the keywords we used for the literature search included, Ghana, intestinal parasites, soil transmitted helminths, helminthiasis, hookworm or *Ancylostoma duodenale* or *Necator americanus*, roundworm or *Ascaris lumbricoides,* whipworm or *Trichuris trichiura,* threadworm or *Strongyloides stercoralis* and pinworm or *Enterobius vermicularis.*

**Search approach:** we entered each keyword or term independently into each search engine of the selected database using the syntax: “term or keyword” AND “Ghana”. The search was systematically carried out to ensure each term was used. For maximum accuracy of results, we performed the search repeatedly on different days within the study period and from the various databases. From the results of each search we first read through the titles and abstracts of the publications and examined them for relevance to soil transmitted helminths in Ghana and the period under review. We selected and downloaded full texts of relevant papers and carefully read through each one for eligibility for inclusion in the review. We also used HINARI search and services of local university libraries to obtain full text articles which were not freely available on the websites. Additionally, we studied linked publications for relevance and eligibility for inclusion in the review. Articles that met the eligibility criteria were included in the review.

**Material processing and analysis:** using the author name, the title and date of paper, we excluded duplicate papers. With thorough reading we extracted relevant data from the full texts and entered them in Microsoft Excel spreadsheet. We extracted the author(s) name, study type, date, site, population and sample size. Others included are method of laboratory analysis, type of STHs and prevalence found as well as factors reported to be associated with the infections within study sites. We summarized the data in tables and presented the findings ensuring that the objectives of the review are achieved.

## Current status of knowledge

**Figure 1:**
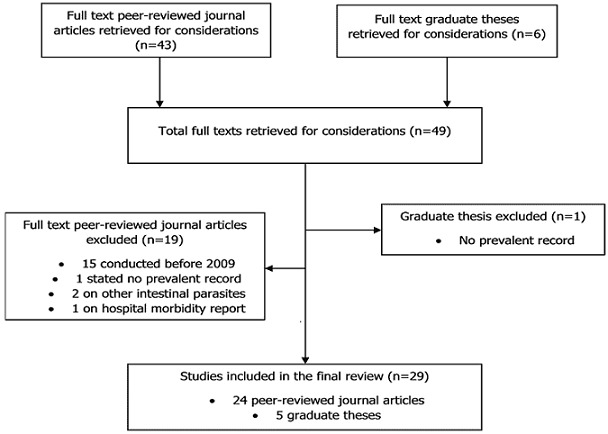
Literature selection procedure flow chart

**Studies under review:** we retrieved a total of 43 full-text articles from peer-reviewed journals and six (6) theses from university repositories within the ten-year period under review. Of this, 29 consisting of 24 published works in peer reviewed journals and four (4) graduate theses from Ghanaian university repositories and one (1) from Yale university scholarly publishing digital platform satisfied the eligibility criteria. Nineteen journal papers were excluded; 15 of the studies were conducted before 2009, two were on parasitic infections other than STHs, one did not state the prevalent records and one was hospital morbidity reports (Figure 1). Table 1, Table 1 (suite), Table 1 (suite 1), Table 1 (suite 2), Table 1 (suite 3) show summaries of 24 studies on soil transmitted helminths conducted in various regions of Ghana from 2009 to 2018 published in peer review journals and Table 2 show five theses that were included in the review. All were descriptive cross-sectional studies with minimum sample size of 83 among pregnant women and highest sample size of 2400 among school children.

**Table 1:** Studies on soil transmitted helminths in Ghana, published in peer reviewed journals in 2009-2018

Authors/ Date/ Study Site	Study Population & Sample Size	Study Setting/ Region/ Ecological zone	Laboratory sample analysis method	Hookworm (Necator & Ansyclostoma) prevalence %	Roundworm (Ascaris) prevalence %	Whipworm (Trichuris) prevalence %	Threadworm (Strongyloides) prevalence %
Humphries *et al.* 2013; Kintampo North Municipality	Children; 6-11years (279)	Rural BAR, FST	Kato-Katz	39.1	none detected	none detected	none detected
Tay *et al.* 2013, KNUST Hospital, Kumasi	Pregnant women; 16-45 years (380)	Urban AR, DF	Direct wet mount	none detected	0.53	0.26	0.53
Adams and Lawson. 2014, KNUST Campus, Kumasi	Food vendors, 10-70 years (140)	Urban AR, DF	Direct wet mount, iodine preparation, and concentration technique	17.8	37.1	0.7	2.9
Boye *et al.* 2014, CRH and UCCH	Pregnant women; 16-45 years (83)	Peri Urban, CR, CS	Direct smear and formol-ether concentration	none detected	10.8	6	3.6
Egbi *et al.* 2014; Adaklu-Anyigbe	Children; 6-12 years (143)	Rural VR, GS	Kato-Katz	9.8	none detected	none detected	none detected

WS- wet season; DS- dry season; AR -Ashanti region; BAR-Brong Ahafo region; NR-Northern Region; VR-Volta region; GAR- Greater Accra region; FST-Forest Savanna Transition; GS- Guinea Savanna; CS-Coastal Savanna; DF- Deciduous Forest

**Table 1 (suite):** Studies on soil transmitted helminths in Ghana, published in peer reviewed journals in 2009-2018

Authors/ Date/ Study Site	Study Population & Sample Size	Study Setting/ Region/ Ecological zone	Laboratory sample analysis method	Hookworm (Necator & Ansyclostoma) prevalence %	Roundworm (Ascaris) prevalence %	Whipworm (Trichuris) prevalence %	Threadworm (Strongyloides) prevalence %
Ofosu *et al.* 2014, Kwahu West Municipality	School children (286)	Rural ER DF	Kato Katz	0.7	3.8	1.4	none detected
Tay *et al.* 2014; Atwima Nwabiegya district	Food vendors (600)	Rural and Urban AR, DF	Direct wet mount, formol-ether concentration and flotation	7	none detected	none detected	21
Agboli *et al.* 2015, KNUST Hospital	Pregnant women (380)	Urban AR, DF	Direct wet mount	none detected	0.5	0.3	0.5
Dankwa *et al.* 2015; Cape Coast Metropolis	School children, 6-17 years (230)	Rural CR, CS	Direct wet mount and formol-ether concentration techniques	3.9	3	1.7	1.7
Danikuu *et al.* 2015; Tamale Metropolitan Assembly	Street food vendors; 15-60 years (150)	Urban, NR GS	Direct smear and formol-saline concentration	none detected	none detected	none detected	5.3

WS- wet season; DS- dry season; AR -Ashanti region; BAR-Brong Ahafo region; NR-Northern Region; VR-Volta region; GAR- Greater Accra region; FST-Forest Savanna Transition; GS- Guinea Savanna; CS-Coastal Savanna; DF- Deciduous Forest

**Table 1 (suite 1):** Studies on soil transmitted helminths in Ghana, published in peer reviewed journals in 2009-2018

Authors/ Date/ Study Site	Study Population & Sample Size	Study Setting/ Region/ Ecological zone	Laboratory sample analysis method	Hookworm (Necator & Ansyclostoma) prevalence %	Roundworm (Ascaris) prevalence %	Whipworm (Trichuris) prevalence %	Threadworm (Strongyloides) prevalence %
Duedu *et al.* 2015; Psychiatric Hospital, Accra	Psychiatric patients, 25-60 (111)	Urban, GAR CS	Direct wet mount, concentration and Ziehl–Neelsen stain	none detected	0.9	2.7	0.9
Duedu *et al.* 2015; Osu Orphanage Accra	Orphans, 5-22 years (101)	Urban GAR, CS	Direct wet mount and formol-ether concentration	1	5	1	2
Tandoh *et al.* 2015; Bongo District, UER	Non-enrolled (145) & enrolled (163) school children (308)	Rural UER, GS	Sedimentation technique and Kato Katz	0.6 (enrolled)	none detected	none detected	none detected
Amoah *et al.* 2016; Kumasi	Vegetable farmers; WS=(165), DS=(127)	Rural, AR DF	Formol-ether concentration	12.73WS, 4.72DS	15.77WS, 11.02 DS	none detected	none detected
Ayeh-Kumi *et al.* 2016; South-Tongu District	School children, 6-13 years (404)	Rural, VR FST	Formol-ether concentration	none detected	none detected	none detected	none detected

WS- wet season; DS- dry season; AR -Ashanti region; BAR-Brong Ahafo region; NR-Northern Region; VR-Volta region; GAR- Greater Accra region; FST-Forest Savanna Transition; GS- Guinea Savanna; CS-Coastal Savanna; DF- Deciduous Forest

**Table 1 (suite 2):** Studies on soil transmitted helminths in Ghana, published in peer reviewed journals in 2009-2018

Authors/ Date/ Study Site	Study Population & Sample Size	Study Setting/ Region/ Ecological zone	Laboratory sample analysis method	Hookworm (Necator & Ansyclostoma) prevalence %	Roundworm (Ascaris) prevalence %	Whipworm (Trichuris) prevalence %	Threadworm (Strongyloides) prevalence %
Forson *et al.* 2017, Odododiodio constituency	School children, 2-9 years (300)	Mixed GAR, CS	Direct wet mount and formol-ether concentration	none detected	1	none detected	0.3
Humphries *et al.* 2017, Kintampo North Municipality (contiguous communities)	School age children, 6-13 years (140)	Rural, BAR FST	Kato–Katz	59	not examined	not examined	not examined
Mirisho *et al.* 2017, Princess Marie children Hospital	Children, 0-10 years (225)	Urban GAR, CS	Direct wet mount	10.22	7.11	none detected	none detected
Orish *et al.* 2017, Ho municipal, Adaklu and Agotime-Ziope districts	School children, 6-14 years (550)	Rural, VR FST	Direct wet mount	0.91	1.27	none detected	none detected
Tay *et al.* 2017; Dangme East District	Pregnant women, 15-49 years (375)	Rural, GAR, CS	Formol-ether concentration	4	8.5	5.9	1.9

WS- wet season; DS- dry season; AR -Ashanti region; BAR-Brong Ahafo region; NR-Northern Region; VR-Volta region; GAR- Greater Accra region; FST-Forest Savanna Transition; GS- Guinea Savanna; CS-Coastal Savanna; DF- Deciduous Forest

**Table 1 (suite 3):** Studies on soil transmitted helminths in Ghana, published in peer reviewed journals in 2009-2018

Authors/ Date/ Study Site	Study Population & Sample Size	Study Setting/ Region/ Ecological zone	Laboratory sample analysis method	Hookworm (Necator & Ansyclostoma) prevalence %	Roundworm (Ascaris) prevalence %	Whipworm (Trichuris) prevalence %	Threadworm (Strongyloides) prevalence %
Adu-Gyasi *et al.* 2018, Kintampo North Municipality and South District	General, 1-96 years (1568)	Rural, BAR FST	Direct wet mount and formol-ether concentration	12.1	1.5	0.8	0.9
Forson *et al.* 2018, Accra (overcrowded urban slums)	School children, 2–9 years (300)	Urban, GAR CS	Direct wet mount and formol-ether concentration	none detected	1	none detected	0.33
Sam *et al.* 2018, Kassena-Nankana East and West Districts. UER	School children, 5-15 years (394)	Rural, UER, GS	Direct wet mount and formol ether concentration	0.25 (direct wet mount) 3.30 (formol ether concentration)	0.00 (direct wet mount) 1.02 (formol Ether concentration)	0.00 (direct wet mount) 0.00 (formol ether concentration)	2.54 (direct wet mount) 5.08 (formol ether concentration)
Squire *et al.* 2018, Shai Osudoku, Kpong, Katamanso, Awutu Senya, Komenda Edina Eguafo Abirem, North Tongu and Central Tongu Districts	Livestock farmers (95)	Rural, GAR, CR & VR, CS	DNA and formol ether concentration	13.7	none detected	none detected	none detected

WS- wet season; DS- dry season; AR -Ashanti region; BAR-Brong Ahafo region; NR-Northern Region; VR-Volta region; GAR- Greater Accra region; FST-Forest Savanna Transition; GS- Guinea Savanna; CS-Coastal Savanna; DF- Deciduous Forest

**Table 2:** Soil transmitted helminths in Ghana from student theses, 2009-2018

Authors/ Date/ Study Site	Study Population & Sample Size	Study Setting/ Region/ Ecological zone	Laboratory sample analysis method	Hookworm (Necator & Ansyclostoma) prevalence %	Roundworm (Ascaris) prevalence %	Whipworm (Trichuris) prevalence %	Threadworm (Strongyloides) prevalence %
Apuusi Clara, 2012 (thesis); Tono Dam, Kassena Nankana District	General, 5-75 years (327)	Rural, UER, GS	Formol ether concentration	0.9	none detected	none detected	none detected
Tetteh Philip, 2012 (thesis); Kumasi Metropolis	School children 5-12years (2400)	Urban, AR, DF	Direct wet mount and formol ether concentration	1.54	3.88	none detected	0.88
Frimpong Francis Kwabena, 2013 (thesis); Bonsaaso cluster, Amansie West District	Pregnant women, 15-41years (186)	Peri-urban, AR, DF	Formol ether concentration	53	14	none detected	16
Jaske Erin Leah, 2014 (Thesis), Kintampo North Municipality	Children. 7-12 years (178)	Rural, BAR, FST	Kato-Katz technique	32.02	none detected	1.12	none detected
Badiwon Charles, 2015 (thesis); Pru and Atebubu District	School children, 1-20 years (659)	Rural, BAR, FST	Direct wet mount and formol ether concentration	2.9A, 2.6P	0.8A, 0.7P	none detected	0.8A, 0.0P

WS- wet season; DS- dry season; AR -Ashanti region; BAR-Brong Ahafo region; NR-Northern Region; VR-Volta region; GAR- Greater Accra region; FST-Forest Savanna Transition; GS- Guinea Savanna; CS-Coastal Savanna; DF- Deciduous Forest

We found studies that were conducted in eight out of the ten administrative regions of the country. Most of the studies were conducted in Ashanti (6), Greater Accra (5) and Brong-Ahafo (5) regions. No research work was reported from the Western and Upper West regions of the country. Nine (9) of the studies were conducted in Coastal savanna, eight (8) in Decidual forest, seven (7) in Forest-savanna transitional and five (5) in Guinea savanna ecological zones. No work was done in the wet Evergreen (part of Western region) and Sudan savanna (part of Upper West region) ecological zones (Table 1, Table 1 (suite), Table 1 (suite 1), Table 1 (suite 2), Table 1 (suite 3), Table 2). Sixteen, nine (9) and four (4) studies were carried out in rural, urban and peri-urban populations respectively. Majority of the studies were conducted among children (16) and pregnant women (5).

**Types of soil transmitted helminths:** as indicated in Table 1, Table 1 (suite), Table 1 (suite 1), Table 1 (suite 2), Table 1 (suite 3), Table 2, the four most common soil transmitted helminths reported in Ghana over the ten-year period of 2009-2018, were hookworm, roundworm, threadworm, and whipworm. Eighteen and seventeen of the twenty-five studies reviewed reported hookworm and roundworm infections respectively. Threadworm and whipworm were third and fourth most frequently reported STHs within the period under review. Very rarely pinworm was reported. The reports on these infections were from different regions and ecological zones of the country. Other intestinal parasites reported included *Schistosoma mansoni, Entamoeba histolytica, Taenia solium, Hymenolepis nana*, and *Giardia lamblia.*

**Geopolitical distribution of the STHs:** the review showed widespread distribution of STHs infections in Ghana. Within the period under review, the infections have been reported from all the ecological zones and all but one administrative region, where studies were conducted in the country. Kintampo district of Brong-Ahafo region was the most endemic area of STHs with hookworm being the most commonly reported. Low prevalence of 0.6-12.1% hookworm infections was also recorded from studies in Upper East, Central, Eastern, Ashanti and Greater Accra and parts of Volta regions (Table 1, Table 1 (suite), Table 1 (suite 1), Table 1 (suite 2), Table 1 (suite 3), Table 2). Roundworm and threadworm were also wide spread in the country.

**Prevalence and populations affected:** soil transmitted infections have been reported among both adults and children. Children were the population most studied. Fifteen of the studies were among school age children and one among younger children in an orphanage. The highest STHs prevalence indicated was hookworm infection of 59% among children of 6-13 years old in the Brong Ahafo region of the forest savanna transition ecological zone [28]. Humphries *et al.* in their studies conducted among children in the area (Kintampo North Municipality) in 2013 recorded hookworm infection prevalence of 39.1% with 82.6% of affected children having light infection [29]. In a similar study conducted in 2017 by the same author, hookworm infection with prevalence of 59% was recorded with light hookworm infection (<1000 epg) of 96% among infected children [28]. Only 4% had moderate infection (2,000-4,999 epg) [28]. In a recent study (2018) among general population aged 1-96 years in the same area, Adu-Gyasi *et al.* recorded 12.1% of hookworm infection [30]. About 55.4%, of those infected had heavy infection, 36.9% had light infection and 7.7% had moderate infection [30]. From the general populations in the two reports, we observed that children below 16 years of ages had the highest prevalence of hookworm infection [30, 31]. Also, we observed that each of hookworm, roundworm, whipworm and threadworm infections each with prevalence greater than 10% were recorded among children who were less than 8 years old [30].

Of the four published research papers conducted among pregnant women, one recorded hookworm with prevalence of 4% [32] and three none [33-35]. Other soil transmitted helminth infections had been recorded from some of the studies done among the population of pregnant women but were of low prevalence (Table 1, Table 1 (suite), Table 1 (suite 1), Table 1 (suite 2)). Additionally, the thesis done among pregnant women stated a prevalence of 53% of hookworm infections [36]. Out of the 53% infected with hookworm, 84% have light infection (1-999 epg), 12% with moderate infection (1000-3999 epg) and 4% with heavy infection (4,000 epg) [36]. Some studies done among children in Accra metropolitan and South Tongu areas [37, 38], adult psychiatric patients in Accra [39], and among adult street food vendors in Tamale metropolis [40] also recorded no hookworm infection. Two studies were conducted among farmers (one among cattle farmers in the Greater Accra, Central and Volta regions of coastal savanna zones altogether while the other one was conducted among vegetable farmers in Ashanti region of deciduous forest).

From the study conducted among vegetable farmers, we observed higher prevalence of hookworm and roundworm in the wet season (12.77% hookworm and 15.77% roundworm) as compared to dry season (4.72% hookworm and 11.02% roundworm) (Table 1 (suite 1)). Also, from the study conducted among cattle farmers from the three regions, majority of the farmers had hookworm (13.7%) infections. We also observed from the study that 9.5% of the farmers had Trichostrongyle infections. Three studies identified and included in this review were among food vendors. One was conducted among street food vendors in the northern region of Guinea savanna zone. From this study, we observed a prevalence record of 5.3% of threadworm infection and no other STHs [40] (Table 1 (suite)). The two other studies were conducted in the Deciduous Forest Zone of Ashanti region. One of the two studies conducted in the Ashanti region, recorded 21% and 7% of threadworm and hookworm respectively (Table 1, Table 1 (suite)). Also found is higher prevalence of STHs among food vendors in rural than urban settings [41]. The second study done at KNUST campus recorded prevalence of *Ascaris lumbricoides* (37.1%), hookworm (17.9%), *Strongyloides stercoralis* (2.9%) and *Trichuris trichiura* (0.7%). About 1.4% had a combination of *Strongyloides stercoralis* and *Trichuris trichiura* infections [42].

**Factors facilitating transmission of the infections:** poor personal hygiene, lack of proper toilet facilities and poor environmental sanitation, poverty and low income, young age and farming activities were identified as factors associated with STHs among the populations studied in the country over the ten-year period under review. Even in the city such as Kumasi, farmers who grow vegetables in dry season use waste to irrigate the crops putting themselves and patrons of their products at getting STH [43]. The use of pit latrine and open defecation in the fields have been reported by most of the studies reviewed including those conducted in Amansie west of Ashanti and Kimtampo in the Brong Ahafo regions [30, 36]. In some areas where communities depend on open small irrigation dams such as Tono in the Upper East region, domestic animals including cattle compete with the people for the water [31]. Scarcity of water and associated poor personal hygiene [30, 44], adults working in infested agricultural fields [43, 45] and children in contact with the infested soil [37] contribute to the high incidence of the infections.

**Ten-year trend of reported infections:** the search revealed no study between the year 2009 and 2011 (Table 1, Table 1 (suite), Table 1 (suite 1), Table 1 (suite 2), Table 1 (suite 3), Table 2). STHs have been reported in the country from 2012 to 2018 and we have observed general downward trend in prevalence and the density of various infections within the period under review. Among children, Tetteh´s study (thesis) in 2012 reported *Ascaris lumbricoides* infection of prevalence 3.88% [46]. In 2012, Apuusi conducted a study (thesis) among the general population, and recorded hookworm prevalence of 0.9% [31]. For the studies conducted in the population of pregnant women, one of the two studies done in 2013 (thesis) by Frimpong had 53% prevalence of hookworm infection [36]. Tay *et al.* in 2013 recorded a prevalence of 0.53% of both *Ascaris lumbriciodes* and *Strongeloides stercoralis* [35]. Humphries *et al.* study conducted in 2013 reported only hookworm infection of 39.1% prevalence [29]. Boye and his colleagues´ study in 2014 reported *Ascaris lumbricoides* of prevalence of 10.8% [34]. Among street food vendors, Adams and Lawson in the year 2014 identified 37.1% of *Ascaris lumbricoides* [42]. Tay *et al.* in the same year (2014) recorded 21% of *Strongyloides stercoralis* [41]. Additionally, Danikuu *et al.* in 2015 recorded 5.3% prevalence of *Strongyloides stercoralis* among street food vendors [40]. Three studies (two published articles and one thesis) were done in 2014 among children. From these, Jaske (thesis) recorded hookworm infection of 32.02% [47], Ofosu *et al.* recorded 3.8% of *Ascaris lumbricoides* [48] and Egbi *et al.* recorded 9.8% of hookworm infection [49]. In Jaske study, those who were lightly infected with the hookworm infection scored the highest infection density of 98.25% [47].

Agboli *et al.* in 2015 reported prevalence of 0.5% each of *Ascaris lumbricoides* and *Striongyloides stercoralis* [33]. Tandoh *et al.* study among both enrolled and non-enrolled school children in the same year of 2015 recorded 0.6% prevalence of hookworm infection among the enrolled school children [50]. Duedu and his colleagues study also conducted in 2015 among psychiatric population recorded 2.7% prevalence of *Trichuris trichiura* [39]. Again, three studies (two published papers and one thesis) were also done in 2015 where Duedu *et al.* recorded *Ascaris lumbricoides* of 5% [51], Dankwa *et al.* recorded hookworm of 3.9% [52], Badiwon recorded hookworm of 2.9% and 2.6% at Atebubu and Pru respectively [53]. In 2016, Amoah and his colleagues recorded 15.8% of *Ascaris lumbricoides* prevalence [43]. Tay *et al.* in 2017 reported *Ascaris lumbricoides* prevalence of 8.5% [32]. In 2018, Squire *et al.* had hookworm infection prevalence of 13.7% [45] whereas Adu-Gyasi and his colleagues (published article) had hookworm prevalence of 12.1% among the general population [30]. Four studies were conducted in 2017 and hookworm infection of 10.22% and 59% were observed to be the highest STHs reported by Mirisho *et al.* [54] and Humphries *et al.* [28] whiles *Ascaris lumbricoides* of 1.27% and 1% were the highest STHs prevalence reported by Orish *et al.* [55] and Forson and his colleagues [38]. Finally, two studies were conducted in 2018 where Forson and his colleagues reported 1% *Ascaris lumbricoides* [44] and Sam *et al.* reported *Strongyloides stercoralis* prevalence of 2.54% and 5.08% (with direct wet mount and formol ether concentration techniques) as the highest among the STHs [56].

Our review indicates paucity of research work on soil transmitted helminths in Ghana over the ten-year period of 2009 - 2018. This seemly lack of research interest in these infections in the country is consistent with WHO declaration of STHs as neglected tropical diseases. In view of limited number and coverage of studies reviewed, our findings may not represent the true burden of STHs in the country. However, the persistence of the infections in regions of the country that have been known to be endemic to these infections calls for evaluation of the effectiveness of local prevention and control measures [16]. The general downward trend in the prevalence and the density of the infections recorded over the period offers some hope of eventual control and elimination of these diseases in the country. The findings also indicate the need for further research into STHs and other neglected tropical diseases in Ghana. Finding hookworm as the most prevalent STHs in the country is quite worrisome since it is associated with significant morbidity of children and pregnant women [28]. The widespread prevalence of the infections implies soil contamination from open defecation due to lack of standard toilet facilities that are hallmarks of overall poor socioeconomic status of communities and individuals. The observation raises serious questions about the effectiveness of national efforts at improving the standard of living of people and the success of control programs [11, 45].

The zero-hookworm prevalence detected in the studies conducted in urban slam of Accra and adjoining rural district of North Tongu is consistent with the observation that Greater Accra and Volta Regions are among the regions classified as low incidence zones of helminths infections in Ghana. The observation could be due to accessibility to STHs control programs and general improvement in environmental sanitation in these areas. Sociocultural practices of the indigenes in areas with low prevalence of STHs need to be studied to establish whether there are any local best practice behaviours that need to be developed and adopted in other areas to help prevent the infections. The review confirms that parasitic infection profile could vary from region to region and even from urban slams to rural areas. While the South Tongu study recorded no STHs, it identified urinary schistosomiasis infections [37].

Similarly, studies done in Greater Accra region recorded no hookworm but roundworm and threadworm infections [38, 44]. Also, the observed frequency of roundworm and whipworm infections in the studies done among children and the general population included in this review is an indication of the need to include both low and high endemic regions in the control programs. We also observed similar prevalence records of STHs among school children and general population from the review of theses presented at university repositories. The pattern of observations is not different from the findings of studies published in peer review journals. This demonstrates the importance of having good institutional repository of students´ research work majority of which, do not get published in peer review journals due to weak local publication culture. These theses could be immensely useful in systematic review and meta-analysis. Finally, we had identified STHs infection prevalence from the two studies done among inmates (orphanage home and psychiatric institution) in the greater Accra region (Table 1 (suite 1)). The observed STHs prevalence record among these populations with special need could be linked to overcrowding nature mostly seen in these institutions that can easily cause the spread of these infections. The need to include these populations in surveillance programs should strongly be considered.

**Limitations:** our study has some limitations that must be taken into consideration. Almost all the studies with the exception of one included in this review made use of basic microscopy techniques for detecting the parasitic infections that to some extent may be less sensitive compared to molecular techniques. Additionally, some of the STHs such as *S. stercoralis* and *E. vermicularis* need specialised laboratory methods for detection, but the studies did not mention the use of such techniques. This could mean that the studies under detected those parasites. Other limitations include small sample size, use of questionnaires not validated.

## Conclusion

We observed low rate of published research work on STHs infections in Ghana. The four most common STHs known in Ghana are still prevalent among all the high-risk populations of children, pregnant women and farmers. Hookworm and roundworm remain the most prevalent of infection recorded in the studies. The highest hookworm infections recorded were from studies done in the forest savanna ecological zone of Brong Ahafo Region, well known for farming activities in Ghana. The prevalence each of the infections among all the high-risk populations within the ten-year period is low and has shown general downward trend. Thus, STHs control intervention programs in the country are having some positive impact. However further research is required in the various regions of the country to support effective development of control programs in the country.

### What is known about this topic

- Soil transmitted infections are worldwide with highest prevalence occurring in sub-Saharan Africa;
- The infections are associated with high mobility and mortality and as such have been targeted for control and elimination by WHO;
- Children, pregnant women and farmers are populations that are mostly at risk of acquiring STHs.

### What this study adds

- Paucity of published research work on soil transmitted helminth infections in Ghana;
- Lack of research interest in soil transmitted helminth infections in Ghana;
- Patterns of STHs prevalence record from theses presented at university repositories are similar to those published in peer review journals.

## Competing interests

The authors declare no competing interests.

## References

[1] Knopp S, Steinmann P, Keiser J, Utzinger J (2012). Nematode Infections. Soil-Transmitted Helminths and Trichinella. Infect Dis Clin NA.

[2] Pullan RL, Brooker SJ (2012). The global limits and population at risk of soil-transmitted helminth infections in 2010. Parasites and Vectors.

[3] World Health Organization Summary of Global Update on Preventive Chemotherapy Implementation in 2015.

[4] Tikasingh ES, Chadee DD, Rawlins SC (2011). The control of hookworm disease in Commonwealth Caribbean countries. Acta Trop.

[5] Hotez PJ, Alvarado M, Basanez M-G, Bolliger I, Bourne R, Boussinesq M (2014). The Global Burden of Disease Study 2010: Interpretation and Implications for the Neglected Tropical Diseases. PLoS Negl Trop Dis.

[6] Keiser J, Utzinger J (2008). Efficacy of Current Drugs Against Soil-Transmitted Helminth Infections: Systematic Review and Meta-Analysis. JAMA.

[7] Ghana Health Service (GHS) Master Plan for Neglected Tropical Diseases Programme, Ghana (2016-2020).

[8] World Health Organization (2017). Guideline: Preventive Chemotherapy to Control Soil-Transmitted Helminth Infections in at-Risk Population Groups.

[9] Brooker S, Kabatereine NB, Smith JL, Mupfasoni D, Mwanje MT, Ndayishimiye O (2009). An updated atlas of human helminth infections: the example of East Africa. Int J Health Geogr.

[10] Salazar-Castanon VH, Legorreta-Herrera M, Rodriguez-Sosa M (2014). Helminth Parasites Alter Protection against Plasmodium Infection. Biomed Res Int.

[11] Hotez P (2008). Hookworm and Poverty. Ann N Y Acad Sci.

[12] Awasthi S, Bundy DAP, Savioli L (1999). Helminthic infections. BMJ.

[13] World Health Organization (2006). Preventive Chemotherapy in Human Helminthiasis: Coordinated Use of Anthelminthic Drugs in Control Interventions: A Manual for Health Professionals and Programme Managers.

[14] Ansell J, Guyatt H, Hall A, Kihamia C, Kivugo J, Ntimbwa P (1997). The reliability of self-reported blood in urine and schistosomiasis as indicators of Schistosoma haematobium infection in school children: a study in Muheza District, Tanzania. Trop Med Int Health.

[15] Partnership for Child Development (1999). The cost of large-scale school health programmes which deliver anthelmintics to children in Ghana and Tanzania. Acta Trop.

[16] USAID END Neglected Tropical Diseases in Africa, Ghana.

[17] Ghana Statistical Service (GSS) 2010 Population and Housing Census Report: Urbanisation.

[18] Brannan M, Hygstedt E, Lindberg J Final Report: Deworm Ghana Program 2012.

[19] Moher D, Liberati A, Tetzlaff J, Altman DG (2009). Preferred reporting items for systematic reviews and meta-analyses: the PRISMA statement. BMJ.

[20] Stanturf JA, Warren ML, Charnley S, Polasky SC, Goodrick SL, Armah F (2011). Ghana climate change vulnerability and adaptation assessment.

[21] Ghana Statistical Service (GSS) Provisional 2017 Annual Gross Domestic Product: April 2018 Edition.

[22] Ghana Living Standard Survey Round 7 (GLSS7). Poverty Trends in Ghana 2005-2017.

[23] World Bank Ghana Education Statistics.

[24] UNICEF (2015). The UNICEF Ghana internal statistical bulletin.

[25] Abdul-Rahman L, Agble R Review of school health and nutrition interventions and mapping of existing programmes in Ghana. 2012.

[26] USAID (2017). Ghana National Anemia Profile.

[27] World Health Organization WHO recommendations on antenatal care for a positive pregnancy experience.

[28] Humphries D, Nguyen S, Kumar S, Quagraine JE, Otchere J, Harrison LM (2017). Effectiveness of Albendazole for Hookworm Varies Widely by Community and Correlates with Nutritional Factors: A Cross-Sectional Study of School-Age Children in Ghana. Am J Trop Med Hyg.

[29] Humphries D, Simms BT, Davey D, Otchere J, Quagraine J, Terryah S (2013). Hookworm Infection among School Age Children in Kintampo North Municipality, Ghana: Nutritional Risk Factors and Response to Albendazole Treatment. Am J Trop Med Hyg.

[30] Adu-Gyasi D, Asante KP, Frimpong MT, Gyasi DK, Iddrisu LF, Ankrah L (2018). Epidemiology of soil transmitted Helminth infections in the middle-belt of Ghana, Africa. Parasite Epidemiol Control.

[31] Apuusi CA (2012). Helminth and Plasmodium falciparum infections among inhabitants of the Tono rrigation catchment area in the Upper East Region.

[32] Tay SCK, Nani EA, Walana W (2017). Parasitic infections and maternal anaemia among expectant mothers in the Dangme East District of Ghana. BMC Res Notes.

[33] Agboli E, Tay SCK, Obirikorang C, Aidoo EY (2015). Malaria and intestinal parasites in pregnant and non-pregnant women: a comparative study at the University Hospital, Kumasi-Ghana. Journal of Medical and Biomedical Sciences.

[34] Boye A, Pappoe F, Anto EO (2014). Is There an Association Between Helminthiasis and Anemia in Pregnancy? A Test Case of Pregnant Women Attending Two Hospitals in Cape Coast. Journal of Basic & Applied Sciences.

[35] Tay SCK, Agboli E, Abruquah HH, Walana W (2013). Malaria and Anaemia in Pregnant and Non-Pregnant Women of Child-Bearing Age at the University Hospital. Journal of Medical Microbiology.

[36] Frimpong FK (2013). Soil Transmitted Helminths (STH) and Schistosomiasis among Pregnant women attending antenatal clinic: A case study of the Bonsaaso cluster in the Amansie West District of Ashanti Region.

[37] Ayeh-Kumi PF, Addo-Safo K, Attah SK, Tetteh-Quarcoo PB, Obeng-Nkrumah N, Awuah-Mensah G (2016). Malaria, helminths and malnutrition: a cross sectional survey of school children in the South Tongu district of Ghana. BMC Res Notes.

[38] Forson AO, Arthur I, Olu Taiwo M, Glover KK, Pappoe Ashong PJ, Ayeh Kumi PF (2017). Intestinal parasitic infections and risk factors: a cross-sectional survey of some school children in a suburb in Accra, Ghana. BMC Res Notes.

[39] Duedu KO, Karikari YA, Attah SK, Ayeh Kumi PF (2015). Prevalence of intestinal parasites among patients of a Ghanaian psychiatry hospital. BMC Res Notes.

[40] Danikuu FM, Azikala O, Baguo FM (2015). Faeco-oral parasitic infection in street food vendors in Tamale, Ghana. Journal of Medical and Biomedical Sciences.

[41] Tay SCK, Akpatason BE, Basing AW (2014). Prevalence of intestinal parasitic infections among food vendors in the Atwima Nwabiegya district in the Ashanti region of Ghana. Sci J Public Heal.

[42] Adams AS, Lawson BW (2014). Prevalence of Ascaris lumbricoides among food vendors on a University Campus in Ghana. Journal of Science and Technology.

[43] Amoah ID, Abubakari A, Stenstro TA, Abaidoo C, Seidu R (2016). Contribution of Wastewater Irrigation to Soil Transmitted Helminths Infection among Vegetable Farmers in Kumasi, Ghana. PLoS Negl Trop Dis.

[44] Forson AO, Arthur I, Ayeh-Kumi PF (2018). The role of family size, employment and education of parents in the prevalence of intestinal parasitic infections in school children in Accra. PLoS One.

[45] Squire SA, Yang R, Robertson I, Ayi I, Squire DS, Ryan U (2018). Gastrointestinal helminths in farmers and their ruminant livestock from the Coastal Savannah zone of Ghana. Parasitol Res.

[46] Tetteh P (2012). A comparative study of intestinal parasitic infection and associated risk factors among primary school children in six neighbouring communities in Kumasi, Ghana: Ayigya, Kentinkrono, Aboabo, Manhyia, Gyinyase And Kyirapatre.

[47] Jaske EL (2014). An assessment of hookworm infection and albendazole treatment failure among children ages 7-12 in Kintampo North Municipality, Ghana.

[48] Ofosu HA, Ako-Nnubeng IT (2014). The Impact of the School Based Deworming Program on Education in the Kwahu West Municipality of Ghana. Journal of Environment and Earth Science.

[49] Egbi G, Steiner-asiedu M, Kwesi FS, Ayi I, Ofosu W, Setorglo J (2014). Anaemia among school children older than five years in the Volta Region of Ghana. Pan Afr Med J.

[50] Tandoh MA, Steiner-asiedu M, Otchere J, Daisieet LA, Appawu MA, Wilson MD (2015). Helminthiasis burden and nutritional status of non-enrolled school-aged children in irrigated farmimg communities in Bongo District, Ghana. Euro J Exp Bio.

[51] Duedu KO, Peprah E, Anim-Baidoo I, Ayeh-Kumi P (2015). Prevalence of Intestinal Parasites and Association with Malnutrition at a Ghanaian Orphanage. Lib Acad.

[52] Dankwa K, Kumi RO, Ephraim RKD, Adams L, Amoako-Sakyi D, Essien-Baidoo S (2015). Intestinal Parasitosis among Primary School Pupils in Coastal Areas of the Cape Coast Metropolis, Ghana. IJTDH.

[53] Badiwon C (2015). Assessment of the impact of 20 years of mass drug administration with ivermectin on the prevalence of Onchocerca volvulus and other soil transmitted helminth infections in children in the Pru and Atebubu districts in Ghana.

[54] Mirisho R, Neizer ML, Sarfo B (2017). Prevalence of Intestinal Helminths Infestation in Children Attending Princess Marie Louise Children's Hospital in Accra, Ghana. Journal of Parasitology Research.

[55] Orish VN, Ofori-amoah J, Amegan-aho KH (2017). Low Prevalence of Helminth Infections among Primary School Children in the Volta Region of Ghana. AJMAH.

[56] Sam Y, Edzeamey FJ, Frimpong EH, Ako AK, Appiah-Kubi K (2018). Prevalence of Soil-Transmitted Helminths among School Pupils in the Upper East Region of Ghana Using Direct Wet Mount Technique and Formol-Ether Concentration Technique. IJTDH.

